# Impact of living patterns and social participation on the health vulnerability of urban and rural older persons in Jiangsu Province, China

**DOI:** 10.1186/s12877-025-05797-w

**Published:** 2025-03-05

**Authors:** Juan Zheng, Jianqiang Xu, Yuhang Wu, Daqi Liu

**Affiliations:** School of Management, Xuzhou Medical University, No.209 Tongshan Road, Xuzhou, Jiangsu Province 221004 China

**Keywords:** Living patterns, Social participation, Older adults, Health vulnerability, Urban and rural communities

## Abstract

**Background:**

This study analyzes the influence of living patterns and social participation on the health vulnerability of older people in urban and rural areas and provides a reference for addressing this vulnerability.

**Methods:**

A total of 3500 participants aged 60 years and above from Jiangsu Province, China, were surveyed. The vulnerability index, which evaluates self-rated health, risk of falling, general pain or discomfort, chronic diseases, emotional characteristics, depression, anxiety, is used to measure health vulnerability. A multiple linear regression model is used to evaluate the effects of living patterns and social participation on health vulnerability.

**Results:**

A certain level of health vulnerability exists among Chinese urban and rural older people. Living with family members has a positive effect on their health. Individuals who live alone have worse health and lower subjective well-being. Social participation significantly reduces the comprehensive levels of general health vulnerability, physical health vulnerability, and mental health vulnerability of older people. For urban older people, living with families reduces the level of physical and mental health vulnerability, whereas living alone significantly increases health vulnerability. Living patterns significantly affect the psychological vulnerability of rural older people. Social participation has an important impact on the health of older people who live alone, especially on the mental health vulnerability of older people who live alone in urban areas.

**Conclusion:**

Living patterns and social participation are important factors that affect the health vulnerability of older people in both urban and rural areas. Social participation has a significant effect on the health of older people who live alone. In particular, for older people who live alone in cities, being socially active can help change their “sedentary” lifestyle, thereby promoting physical and mental health and reducing vulnerability.

## Introduction

As a country with a large population, China entered a period of continuous and rapid population aging in the first half of the 21st century. According to the seventh demographic census, China’s older adult population has reached 260 million, accounting for 18.7% of the total population [1]. In addition to the growth of the aging population, older people cannot avoid a decline in physical function and easily suffer from the impacts of disease, falls, disability and other risks. Coupled with the gradual weakening of the intergenerational support function of the family and a reduction in social participation, older people gradually exhibit physical and mental vulnerability and health problems. Therefore, actively coping with aging and improving their quality of life in old age have become social problems that must be solved.

At present, scholars mostly use a single measurement index to measure the health of older people, which lacks comprehensiveness and objectivity. Health vulnerability is a comprehensive construct that can fully reflect multidimensional health levels [2]. The health vulnerability of older people is derived from the concept of vulnerability. At present, differences in definitions of the concept of vulnerability remain. Existing studies have reached two basic consensuses: one is that the concept of vulnerability is prospective, and the other is that risk is closely related to vulnerability [3–4]. Most scholars generally start from the two aspects of risk exposure and coping ability and believe that health vulnerability is a combination of sensitivity, easy exposure and a lack of health resilience [5]. Based on relevant studies, we believe that the health vulnerability of older people is a state of poor physical and psychological health that is due to an increase in group sensitivity and a decline in the capacity to protect oneself from internal and external risks.

Previous studies that have addressed health vulnerability in older people have often equated “frailty” with “vulnerability” [6]. However, some researchers believe that vulnerability and frailty cannot be understood as the same concept and that vulnerability is not equal to frailty. Vulnerability can lead to frailty, resulting in a decline in physical or mental function [7]. Compared with frailty, vulnerability is prospective and closely associated with risk. Nevertheless, when scholars measure the health vulnerability of older adults, they tend to view frailty as the main indicator. Rockwood [8] and Mitniski [9] proposed constructing a frailty index (FI) to measure the health vulnerability of older people, and this index has been widely used in academic circles. The frailty index condenses the “health deficits” of older people to form a fitted index. There are two main types of assessments: physiologic performance and cumulative deficit [7, 10, 11]. The former type assesses and measures health vulnerability through a standardized assessment scale or questionnaire. The latter type is a health vulnerability indicator that is derived by calculating the ratio of the cumulative number of health deficits to all health indicators [12]. Cumulative deficits are usually assessed by selecting physical, functional, and psychological health indicators, such as self-rated health, physical health, ability to perform daily activities, depression, anxiety, etc. Current scholars have transitioned the measurement of health vulnerability from a purely physical construct to a multidimensional concept that encompasses both objective and subjective evaluations [13, 14]. For example, health vulnerability usually includes two dimensions, physical health and mental health. Physical health indicators usually include objective evaluations of physical diseases and subjective evaluations of self-rated health.

Scholars have also begun to focus on the factors that influence the health vulnerability of older people. The concept of the health vulnerability of older people originated from vulnerability theory. Vulnerability usually refers to a state that is prone to damage that results from social or environmental changes and a lack of resilience. Risk, sensitivity, the natural environment and the social environment are all key factors that affect vulnerability [15, 16], especially socioenvironmental disadvantages (unequal patterns of living and social participation, etc.), which have received extensive attention from scholars. From the perspective of environmental gerontology, older adults’ health outcomes, such as physical function and cognitive capacity, are closely related to the physical environment (housing, community, public spaces, access and quality of health services) and the social environment (support networks). One study showed that housing issues are of fundamental importance to the health and independence of older people in Britain [17]. Some scholars have pointed out that older adults in the community with a lower quality of life are more likely to experience delayed care, which affects health [18], and older people’s families depend on support networks for their health maintenance [19].

In addition, living patterns and social participation are important social and environmental factors, and their impact on health has also been widely studied. Under the influence of the traditional Chinese cultural concept of “The more sons, the more happiness”, most older people prefer to live with their families. However, with the gradual reduction in family size in China, the number of older people who live alone, empty-nest families, and those who live in nursing homes continues to increase. Some scholars believe that older people who live alone have a greater sense of loneliness [20]. Moreover, older people are prone to depression and other mental illnesses [21] and have poorer health than those who live with their families [22]. However, living alone may also motivate older people to engage in more social participation outside of the home [23], and living with families may lead to family conflicts or other negative social interactions that affect their health. In addition, research has suggested that living alone is not a major factor in triggering poor health in older people [24] and that infrequent social interactions and social participation may have a greater impact than living patterns do [25]. Social participation is an important component of the “active aging” strategy. Research has shown that social connections and participation in leisure activities, such as square dancing, have great health benefits [26]. Due to the urban‒rural dual structure, there may be great differences in health status, living patterns and social participation between urban and rural older people. One study suggested that rural older adults have lower social functioning than their urban counterparts and that they need social relationships and support to increase their participation in the community to increase their QOL [27]. Some researchers have focused on the differences between urban and rural living environments and explored the impact of physical activity on the health of urban and rural older people [28]. In addition, one study noted rural‒urban differences in the relationships between the availability of recreational facilities, physical activity (PA), functional health status, and depressive symptoms in older Chinese adults [29].

In summary, previous studies have shown that living patterns and social participation are important factors that influence the health vulnerability of older people. Some scholars have suggested that living patterns may stimulate social participation among older people [23], thus affecting their health; this suggests that the effect of living patterns on the health vulnerability of older people may be mediated by social participation. Although researchers have begun to explore the mediating effects of social participation on living patterns and health vulnerabilities, few studies have investigated the differences in these effects, and their interaction, on the health vulnerability of older people in urban and rural China. Therefore, the aim of this study was to analyze the effects of living patterns and social participation, and their interactions, on health vulnerability in older adults and to explore rural–urban differences. On this basis, this study raises three research questions: What is the health vulnerability level of urban and rural older people? How do living patterns and social participation and their interactions affect the health vulnerability of older persons? Is there a rural–urban difference in this effect? This study provides a basis for addressing the health vulnerability of urban and rural older people and improving their quality of life.

## Methods

### Sampling and inclusion criteria

A survey was conducted that focused on the health and aging of older people in Jiangsu Province, China. A multistage stratified random cluster sampling method was used. There are 13 prefecture-level cities in Jiangsu Province, which are divided into three groups according to economic level (good economy, average economy, poor economy). Two prefecture-level cities were then randomly selected from each group, and a total of 6 prefecture-level city samples were obtained. First, the socioeconomic differences among neighborhoods in the studied cities should be considered. For each city, we also divided urban neighborhoods into three groups (well-developed neighborhoods, generally developed neighborhoods and poorly developed neighborhoods) according to economic development level, geographical location and other factors. Then, 100 older people were randomly selected from each group as the research sample. Second, 2 towns were randomly selected from each prefecture-level city, and 2 villages were randomly selected from each town. The older people in all villages were selected as the investigation sample. The invalid samples were deleted, and 1682 urban samples and 1818 rural samples were ultimately obtained. The inclusion criteria for the survey respondents were (1) age 60 years or older, (2) long-term residence at the survey site for more than 6 months, (3) normal language expression and good communication skills, and (4) voluntary acceptance of the survey. Older people with language barriers were excluded from the analysis. A structured questionnaire was developed to obtain the relevant information. The pre-survey was administered after the completion of the first draft of the questionnaire, and 100 older people were randomly selected from Xuzhou, Jiangsu Province, for face‒to-face interviews to test the logic of the questionnaire and other questions. The questionnaire was subsequently modified and improved according to the problems identified in the investigative process. To ensure the quality of the questionnaire, we carried out reliability and validity analyses. The results show that the reliability and validity of the questionnaire are good.

### Variable definitions and descriptions

The dependent variable in this study was the health vulnerability of older people. Through a literature review, we found that the FI is widely used internationally to measure health vulnerability. The FI contains a wide range of health information and has been widely recognized and applied in gerontology and demography research; it can be understood as evaluating a number of health deficiencies. These deficiencies can be measured physically, functionally, psychologically, etc. There is no uniform standard for the number of health variables included in the FI or for the choice of specific health variables. The results of an empirical study showed that the FI is not sensitive to the selected health variables [30]. In practice, different studies have varied greatly in the number of variables and the selection of specific variables when constructing the FI, but the research conclusions are very similar, indicating that the FI method is very stable.

The present study uses Rockwood’s approach and the international common indicator assignment method [7] and considers the availability of data to build a measurement index system from the two dimensions of physical vulnerability and psychological vulnerability. Physical health vulnerability indicators included self-rated health, risk of falling, general pain or discomfort, and chronic diseases. Mental health vulnerability indicators included emotional characteristics, depression, and anxiety. Depression was measured with the self-rating depression scale (SDS), which contains 20 items. Each item is assigned a value of 1, 2, 3, or 4. First, we sum the scores of the 20 items to obtain X and calculate Y using the formula Y=int (1.25X). The sum of Y is then calculated. Finally, the depression index = the sum of Y/80×100%. Anxiety was measured with the Generalized Anxiety Disorder-7 scale (GAD-7). The GAD-7 consists of seven items. Each item is assigned 0, 1, 2, or 3 points. The higher the GAD-7 score is, the more severe the anxiety is [31]. The specific health vulnerability indicator assignments are shown in Table 1.

To examine the health vulnerability characteristics of older people, this study referred to the established literature [32] and summed the scores of the different dimensional indicators and then divided this sum by the theoretical maximum score to obtain the health vulnerability index, which has a range of 0–1. In this study, the FI was calculated as the sum of scores of all health vulnerability indicators /7 (the sum of the highest scores for the 7 health indicators = 7). A higher score corresponds to more serious health vulnerability. The obtained results reflect the level of health vulnerability of older people.

**Table 1:** Health vulnerability measurement indices of older people and their assignments

Indicator	Definition of indicator	Variable assignment
**Physical health vulnerability indicators**		
Self-rated health	How do you feel about your current physical health?	Great = 0, Good = 0.25, Normal = 0.5, Bad = 0.75, Terrible = 1
Risk of falling	Have you had a fall in the past year?	Yes = 1, No = 0
General pain or discomfort	Have you had general physical pain or discomfort in the last two weeks?	Yes = 1, No = 0
Chronic diseases	Have you had a chronic disease?	Yes = 1, No = 0
**Mental health vulnerability indicators**		
Emotional function	Can you always get over things, no matter what happens to you?	Particularly = 0, Yes = 0.25, Normal = 0.5, No = 0.75, Extremely no = 1
Depression	Geriatric depression scale (depression index)	Index below 50% =0, 50–59% =0.33, 60–69% =0.66, 70% and above = 1
Anxiety	Geriatric anxiety scale (GAD score)	Score of 0–4 = 0, score of 5–9 = 0.33, score of 10–14 = 0.66, score of 15–21 = 1

Source: Authors’ compilation

The independent variable in this study was living patterns. The living patterns of older people were determined through the following questionnaire item: “Who do you currently live with?” There were three main options: living with family, living alone, and living in a nursing institution. The moderating variable was social participation, measured in ten dimensions based on the following question: “Are you currently engaged in/participating in the following activities?” The items included housework, tai chi, square dancing, visiting, planting flowers, reading books and newspapers, raising poultry (livestock), playing cards (mahjong), watching TV and listening to the radio, and organizing activities in the community. The options were as follows: ① do not participate, ② participate occasionally, ③ participate at least once a month, ④ participate at least once a week, and ⑤ participate almost every day. These options are assigned scores ranging from 1 to 5; a higher score indicates greater social participation. We calculated the Cronbach’s alpha for the sum score of social participation (Cronbach’s alpha = 0.722).

The control variables were selected with reference to the relevant literature and included age (continuous variable), gender (male or female), education (elementary school and below, junior or senior high school, junior college and above), marital status (married or single, divorced, widowed, and unmarried), income (logarithmic), registered permanent residence (urban or rural), health care accessibility (mainly geographical accessibility, a home that is more than 1 km from the nearest medical facility is considered inaccessible, and less than 1 km is considered accessible), and the utilization of community-based older care services (living care, home care, home medical care, spiritual comfort, daily shopping, organizational activities, legal assistance, health guidance, and dispute handling). Each service has two options: ① This service has been used (score = 1); ② This service has not been used (score = 0). Finally, all the scores are added together to obtain a total score.

### Procedure and statistical analysis

This study was ethically reviewed and approved by the Ethics Committee of Xuzhou Medical University (XZHMU-2022085). Before administering the survey, we explained the purpose of the study to the respondents, emphasized the confidentiality of the data, ensured the protection of their privacy, and obtained their informed consent. Once the data were collected, they were processed and analyzed using Stata software. Due to the fact that the dependent variable in this study was the health vulnerability score (which is a continuous variable), we used multiple linear regression models to analyze the effects of living patterns and social participation and their interaction on the health vulnerability of older people.

## Results

### Descriptive statistical analysis

The average age of the respondents was 71.78 years (urban: 72.28 years, rural: 71.33 years). The percentage of female older individuals was 55.83% (urban: 52.56%, rural: 58.86%), and that of male older individuals was 44.17% (urban: 47.44%, rural: 41.14%). There were fewer older people with a college education or higher, accounting for only 10.54% (urban: 18.13%, rural: 3.52%). There were more single (including divorced, widowed and unmarried) older people, accounting for 57.89% of the sample (urban: 54.28%, rural: 61.22%). The average community health service utilization score was 2 points (urban: 2.50 points, rural: 1.53 points). The average income value (logarithmic) was 10.31 (urban: 10.99, rural: 9.68). There were fewer older people in urban areas than in rural areas, accounting for 48.40% and 51.60%, respectively. 97% of older persons had good access to health services (urban: 98.51%, rural: 95.60). With respect to living patterns, the majority of older people lived with their families, accounting for 78.57% (urban: 78.83%, rural: 78.33%), followed by those who lived alone, who accounted for 15.91% (urban: 11.47%, rural: 20.02%). The percentage of older people living in nursing institutions was the lowest, accounting for 5.51% (urban: 9.69%, rural: 1.65%). Using a chi-square test, we found that there was a statistically significant difference between urban and rural older living patterns (*P* < 0.001). The average social activity participation score was 21.40 (urban: 22.86, rural: 20.06). The results of a single-factor analysis revealed that there was a statistically significant difference between urban and rural older social participation (*P* < 0.001). The average and standard deviation of the older participants’ self-rated health, risk of falling, general pain or discomfort, chronic diseases, emotional characteristics, depression, and anxiety, as well as the urban‒rural differences among the survey respondents, are detailed in Table 2.

**Table 2:** Variable descriptive analysis results

Variable	Percentage/average value (standard deviation)			Variable	Percentage/average value (standard deviation)		
	Full sample	Urban	Rural		Full sample	Urban	Rural
	*N* = 3500	*N* = 1682	*N* = 1818		*N* = 3500	*N* = 1682	*N* = 1818
Age	71.78 (11.99)	72.28 (11.92)	71.33 (12.05)	Income (logarithm)	10.31 (1.27)	10.99 (0.77)	9.68 (1.32)
Gender				Registered permanent residence			
Male	44.17	47.44	41.14	Urban	48.40	---	---
Female	55.83	52.56	58.86	Rural	51.60	---	---
Education				Health care accessibility			
Elementary school and below	46.31	24.79	66.23	Accessible	97.00	98.51	95.60
Junior or senior high school	43.14	57.07	30.25	Inaccessible	3.00	1.49	4.40
Junior college and above	10.54	18.13	3.52	Living patterns (*P* < 0.001)			
Marriage				Living with families	78.57	78.83	78.33
Married	42.11	45.72	38.78	Living alone	15.91	11.47	20.02
Single	57.89	54.28	61.22	Nursing institution	5.51	9.69	1.65
Community care services	2.00 (2.36)	2.50 (2.69)	1.53 (1.89)	Social participation (*P* < 0.001)	21.40 (7.89)	22.86 (8.37)	20.06 (7.17)
Self-rated health	0.443 (0.282)	0.435 (0.288)	0.450 (0.275)	Emotional characteristics	0.333 (0.289)	0.307 (0.292)	0.357 (0.284)
Risk of fall	0.220 (0.414)	0.245 (0.430)	0.196 (0.397)	Depression	0.348 (0.165)	0.345 (0.179)	0.351 (0.151)
General pain or discomfort	0.183 (0.387)	0.178 (0.383)	0.188 (0.391)	Anxiety	0.195 (0.303)	0.186 (0.301)	0.203 (0.305)
Chronic diseases	0.250 (0.433)	0.287 (0.452)	0.216 (0.412)				

Source: Authors’ compilation

### Measurement of the health vulnerability levels of older people

As shown in Table 3, the comprehensive index of health vulnerability of the surveyed older individuals was 0.282, the physical health vulnerability index was 0.274, and the mental health vulnerability index was 0.291. The comprehensive index of health vulnerability of urban older individuals was 0.283, the physical health vulnerability index was 0.286, and the mental health vulnerability index was 0.279. The comprehensive index of health vulnerability of rural older individuals was 0.275, the physical health vulnerability index was 0.263, and the mental health vulnerability index was 0.299. Previous studies have shown that older people with a health vulnerability index less than 0.25 are considered to have a low level of health vulnerability, whereas those with a score greater than or equal to 0.25 are considered to have a high level of health vulnerability [32]. The results of this study indicate that the surveyed older individuals generally have some degree of health vulnerability problems. There were no statistically significant differences between rural and urban areas in terms of the comprehensive index of health vulnerability of older people (*P* > 0.05). The physical health vulnerability and mental health vulnerability indexes of older people in urban and rural areas were significantly different (*P* < 0.05). The physical health vulnerability of urban older people was greater than that of rural older people, whereas the mental health vulnerability was lower than that of rural older people. The level of health vulnerability of older people is characterized by a certain degree of urban‒rural heterogeneity.

**Table 3:** Health vulnerability index of older people

	Full sample (*n* = 3500)	Urban (*n* = 1682)	Rural (*n* = 1818)	*p*
Comprehensive index of health vulnerability	0.282	0.283	0.275	0.837
Physical health vulnerability index	0.274	0.286	0.263	0.006
Mental health vulnerability index	0.291	0.279	0.299	< 0.001

Source: Authors’ compilation

### Effects of living patterns and social participation on health vulnerability (full sample)

We analyzed the effects of independent variables, such as living patterns and social participation, on the dependent variables, including the comprehensive health vulnerability score, the total physical health vulnerability score, and the total mental health vulnerability score. Because the dependent variables were all continuous, a multiple linear regression model was used for the analysis. Table 4 shows the results of the model estimation for the full sample.

The results of the analysis clearly indicate that, without considering the influence of other factors, compared with older people living in institutions, older people living with their families have lower levels of comprehensive health vulnerability, physical health vulnerability, and mental health vulnerability (decreases of 0.105, 0.068, and 0.038 units, respectively). Compared with older people living in institutions, those living alone have significantly greater comprehensive health vulnerability, physical health vulnerability, and mental health vulnerability (0.424, 0.165, and 0.259 units, respectively). Social participation significantly reduces the comprehensive level of health vulnerability, physical health vulnerability, and mental health vulnerability of older people (by 0.058, 0.029, and 0.029 units, respectively).

In addition, the comprehensive levels of health vulnerability and mental health vulnerability are lower for men than for women. The health vulnerability of older people with an elementary school education or less is greater than that of those with a junior college education or above. The overall levels of health vulnerability and physical health vulnerability of the urban older population are greater than those of the rural older population; however, the mental health vulnerability is less than that of the rural older population. Older people with better access to health care have lower overall levels of health vulnerability, physical health vulnerability and mental health vulnerability.

**Table 4:** Multiple linear regression results (full sample)

Variable (control group)	Comprehensive health vulnerability		Physical health vulnerability		Mental health vulnerability	
	$\:\varvec{\beta\:}$	S.E	$\:\varvec{\beta\:}$	S.E	$\:\varvec{\beta\:}$	S.E
**Living patterns (nursing institution)**						
Living with families	-0.105^*^	0.066	-0.068^*^	0.047	-0.038^*^	0.032
Living alone	0.424^***^	0.101	0.165^*^	0.072	0.259^***^	0.048
Social participation	-0.058^***^	0.003	-0.029^***^	0.002	-0.029^***^	0.001
Age	-0.002	0.002	0.001	0.001	-0.003^**^	0.001
Gender (female)	-0.114^*^	0.046	-0.041	0.033	-0.074^**^	0.022
**Education (elementary school and below)**						
Junior or senior high school	0.029	0.051	-0.008	0.037	0.037	0.025
Junior college and above	0.171^*^	0.081	0.109	0.058	0.062	0.039
Marriage (single)	0.005	0.053	0.032	0.038	-0.027	0.025
Registered permanent residence (rural)	0.186^**^	0.056	0.189^***^	0.040	-0.003	0.027
Income	-0.019	0.021	-0.012	0.015	-0.007	0.010
Health care accessibility (inaccessible)	-0.643^***^	0.130	-0.334^***^	0.093	-0.309^***^	0.062
Community care service	0.011	0.010	0.007	0.007	0.004	0.005
Constant term	4.179^***^	0.269	1.950^***^	0.193	2.229^***^	0.129
R-squared	0.129		0.066		0.154	

^***^P < 0.001, ^**^0.001 ≤ *P* < 0.01, ^*^0.01 ≤ *P* < 0.05

Source: Authors’ compilation

Table 5 shows the results of the analysis of the effect of the interaction of mode of residence and social activity participation on the health vulnerability of older adults. The interaction term between living with family and social activity participation was not statistically significant. However, the interaction term of living alone and social activity participation was statistically significant. When controlling for other variables, the predicted combined level of health vulnerability was 5.433–0.103 (social activity participation) for older adults living alone and 4.150–0.056 (social activity participation) for older adults living in institutionalized care (the reference group). Thus, social activity participation had a greater effect on the composite level of health vulnerability for older adults living alone than for older adults living in institutions. In addition, because the coefficient was negative, increased social activity participation decreased the composite level of health vulnerability of older adults.

When controlling for other variables, the predicted level of mental health vulnerability was 3.071–0.062 (social activity participation) for older adults living alone and 2.183–0.027 (social activity participation) for older adults living in institutions (the reference group). Thus, social activity participation had a greater effect on the mental health vulnerability level of older adults living alone than on that of older adults living in institutions. In addition, because the coefficient was negative, an increase in social activity participation decreased the mental health vulnerability level of older adults. In addition, the effect of the interaction term between living alone and social activity participation on the physical health vulnerability of older adults was not statistically significant (*p* > 0.05).

**Table 5:** Analysis of interactions between living patterns and social participation (full sample)

Variable	Comprehensive health vulnerability	Physical health vulnerability	Mental health vulnerability
	β (S.E)	β (S.E)	β(S.E)
Living with families	-0.008 (0.184) ^*^	-0.097 (0.132) ^*^	0.090 (0.088) ^*^
Living alone	1.283 (0.265) ^***^	0.394 (0.190) ^*^	0.888 (0.127) ^***^
Social participation	-0.056 (0.003) ^***^	-0.029 (0.003) ^***^	-0.027 (0.002) ^***^
Living with families × Social participation	0.005 (0.008)	0.008 (0.006)	-0.003 (0.004)
Living alone × Social participation	-0.047 (0.013) ^***^	-0.013 (0.010)	-0.035 (0.006) ^***^
Other variables	Controlled	Controlled	Controlled
Constant items	4.150	1.966	2.183
Sample number	3500	3500	3500
R-squared	0.133	0.067	0.161

^***^P < 0.001, ^**^0.001 ≤ *P* < 0.01, ^*^0.01 ≤ *P* < 0.05; “Living in nursing institutions” was the control group

Source: Authors’ compilation

### Impact of residence style and social activity participation on health vulnerability (rural‒urban differences)

Table 6 shows the results of the regression analysis of urban‒rural differences in the health vulnerability of older adults. For urban older adults, living with family members decreased the combined level of health vulnerability, physical health vulnerability, and mental health vulnerability compared with living in an institutionalized setting (by 0.038, 0.069, and 0.031 units, respectively). Living alone significantly increased the combined level of health vulnerability, physical health vulnerability, and mental health vulnerability among urban older adults (by 0.423, 0.170, and 0.252 units, respectively). Social activity participation also significantly decreased the combined level of health vulnerability, physical health vulnerability, and mental health vulnerability of urban older adults (by 0.061, 0.029, and 0.032 units, respectively).

For rural older adults, living with family reduced mental health vulnerability, and living alone increased mental health vulnerability, compared with living in an institution. However, the effect of residential mode on the composite level of health vulnerability and physical health vulnerability of rural older adults was not statistically significant (*p* > 0.05). Social participation also significantly reduced the composite level of health vulnerability, physical health vulnerability, and mental health vulnerability of rural older adults (by 0.053, 0.027, and 0.025 units, respectively).

In terms of the individual characteristics of older adults, the combined level of health vulnerability and the level of mental health vulnerability were significantly lower for males than for rural females; however, the effect of gender on the level of health vulnerability of urban older adults was not statistically significant (*p* > 0.05). Compared with older adults with primary education and below, rural older adults with tertiary education and above had increased levels of health vulnerability. However, the effect of educational attainment on the level of health vulnerability of urban older people was not statistically significant (*p* > 0.05). Medical accessibility was shown to reduce the combined level of health vulnerability, physical health vulnerability, and mental health vulnerability of rural older adults, as well as the combined level of health vulnerability of urban older adults; however, the effect on physical health vulnerability and mental health vulnerability of urban older adults was not statistically significant (*p* > 0.05).

**Table 6:** Results of multiple linear regression analysis (urban‒rural differences)

Variable (control group)	Comprehensive health vulnerability		Physical health vulnerability		Mental health vulnerability	
	Urban	Rural	Urban	Rural	Urban	Rural
**Living patterns (nursing institution)**						
Living with families	-0.038^*^ (0.114)	-0.134 (0.079)	-0.069^*^ (0.080)	-0.051 (0.058)	-0.031^*^ (0.056)	-0.083^*^ (0.037)
Living alone	0.423^***^ (0.117)	0.343 (0.238)	0.170^*^ (0.083)	0.182 (0.175)	0.252^***^ (0.057)	0.161^*^ (0.111)
Social participation	-0.061^***^ (0.005)	-0.053^***^ (0.004)	-0.029^***^ (0.003)	-0.027^***^ (0.003)	-0.032^***^ (0.002)	-0.025^***^ (0.002)
Age	0.001 (0.003)	-0.004 (0.002)	0.003 (0.002)	-0.001 (0.002)	-0.002 (0.001)	-0.003 (0.001)
Gender(female)	-0.093 (0.071)	-0.146^*^ (0.060)	-0.038 (0.050)	-0.050 (0.044)	-0.055 (0.035)	-0.096^**^ (0.028)
**Education (elementary school or below)**						
Junior school or high school	0.071 (0.082)	-0.023 (0.066)	-0.024 (0.058)	-0.007 (0.048)	0.094^*^ (0.040)	-0.016 (0.031)
Junior college or above	0.096 (0.107)	0.692^***^ (0.159)	0.033 (0.075)	0.429^***^ (0.117)	0.063 (0.052)	0.264^***^ (0.075)
Marriage (single)	0.082 (0.081)	-0.061 (0.070)	0.088 (0.057)	-0.019 (0.051)	-0.005 (0.040)	-0.041 (0.033)
Income	-0.011 (0.045)	-0.012 (0.023)	0.018 (0.032)	-0.019 (0.017)	-0.028 (0.022)	0.006 (0.011)
Health care accessibility (inaccessible)	-0.546^*^ (0.277)	-0.687^***^ (0.141)	-0.319 (0.195)	-0.340^**^ (0.103)	-0.227 (0.135)	-0.347^***^ (0.066)
Community care service	0.012 (0.013)	0.013 (0.015)	0.004 (0.009)	0.016 (0.011)	0.009 (0.006)	-0.003 (0.007)
Constant items	3.991^***^ (0.583)	4.301^***^ (0.312)	1.630^***^ (0.409)	2.183^***^ (0.229)	2.361^***^ (0.284)	2.117^***^ (0.146)
R-squared	0.137	0.129	0.065	0.068	0.171	0.142

^***^P < 0.001, ^**^0.001 ≤ *P* < 0.01, ^*^0.01 ≤ *P* < 0.05

Source: Authors’ compilation

Table 7 shows the results of the analysis of the differences in the effects of the interaction of living patterns and social participation on the health vulnerability of urban and rural older people. The effect of the interaction term of living with family and social participation on the health vulnerability of urban and rural older people was not statistically significant (*p* > 0.05). The effect of the interaction term of living alone and social participation on the comprehensive level of health vulnerability and the level of mental health vulnerability of urban older people was statistically significant. The effects on the comprehensive level of health vulnerability, physical health vulnerability, mental health vulnerability, as well as the level of physical health vulnerability of the rural older population and the level of physical health vulnerability of the urban older population, were not statistically significant.

As demonstrated by the results, after controlling for other variables the predicted comprehensive level of health vulnerability was 5.145–0.108 (social activity participation) for urban older people living alone and 3.851–0.060 (social activity participation) for those living in institutions (the reference group). Thus, social participation had a greater effect on the comprehensive level of health vulnerability for urban older people living alone than for those living in institutions. In addition, because the coefficient was negative, this result suggested that an increase in social participation decreases the comprehensive level of health vulnerability among urban older people.

After controlling for other variables, the predicted level of mental health vulnerability was 3.191–0.067 (social activity participation) for urban older people living alone and 2.245–0.029 (social activity participation) for those living in institutions (the reference group). Thus, social participation had a greater effect on the mental health vulnerability level of urban older people living alone than on those living in institutions. In addition, because the coefficient was negative, this result suggested that an increase in social participation decreases the mental health vulnerability level of urban older people.

**Table 7:** Analysis of interactions between living patterns and social participation (urban‒rural differences)

Variable	Comprehensive health vulnerability		Physical health vulnerability		Mental health vulnerability	
	Urban	Rural	Urban	Rural	Urban	Rural
Living with families	-0.514 (0.339)	0.203 (0.227)	-0.285 (0.239)	-0.038 (0.166)	-0.229 (0.164)	0.242 (0.106)
Living alone	1.294^***^ (0.305)	0.553 (0.767)	0.345 (0.215)	0.671 (0.563)	0.946^***^ (0.148)	-0.118 (0.359)
Social participation	-0.060^***^ (0.005)	-0.052^***^ (0.005)	-0.030^***^ (0.004)	-0.028^***^ (0.004)	-0.029^***^ (0.002)	-0.024^***^ (0.002)
Living with families × Social participation	0.022 (0.013)	-0.003 (0.011)	0.014 (0.009)	0.004 (0.008)	0.008 (0.006)	-0.008 (0.005)
Living alone × Social participation	-0.048^**^ (0.015)	-0.012 (0.041)	-0.010 (0.011)	-0.028 (0.030)	-0.038^***^ (0.007)	0.016 (0.019)
Other variables	Controlled	Controlled	Controlled	Controlled	Controlled	Controlled
Constant items	3.851	4.284	1.606	2.197	2.245	2.087
Sample number	1682	1818	1682	1818	1682	1818
R-squared	0.145	0.129	0.067	0.069	0.185	0.144

^***^P < 0.001, ^**^0.001 ≤ *P* < 0.01, ^*^0.01 ≤ *P* < 0.05; “Living in nursing institutions” was the control group

Source: Authors’ compilation

## Discussion

We first measured the current status of the health vulnerability of older people. The comprehensive index of the health vulnerability of older people is 0.282, the index of physical health vulnerability is 0.274, and the index of mental health vulnerability is 0.291. Studies have shown that a health vulnerability index greater than or equal to 0.25 is considered to represent a high level of health vulnerability among older people [32]. Therefore, the surveyed older people had some degree of health vulnerability problems, which is consistent with the findings of previous studies [33], and physical health vulnerability problems are more serious than mental health vulnerability problems. The level of health vulnerability of older people is characterized by a certain degree of urban‒rural heterogeneity. The physical health vulnerability of urban older people was greater than that of rural older people, whereas their mental health vulnerability was lower than that of rural older people. Vulnerability theory states that the health vulnerability of older people can be understood as follows: older adults are easily exposed to health risks and will suffer diminished health due to a lack of health repair capability. According to vulnerability theory, the results of this study suggest that elderly individuals in urban areas are more likely to be exposed to physical health risks, which may lead to physical health problems. Rural older people are more likely to be exposed to mental health risks, which can lead to mental health problems. Older adults are particularly inactive and sedentary, posing unique public health challenges [34]; this may be an important reason for the high physical health vulnerability of urban older people. Many elderly people residing in rural areas face greater economic pressure and difficulties in securing their own livelihood because they provide financial support to their children. Therefore, from a psychological perspective, it is easier for them to develop a sense of stress. In addition, rural-dwelling elderly individuals who experience mental health problems often have difficulty finding help because rural communities often lack adequate mental health service providers [35].

By analyzing the effects of living patterns and social participation on the health vulnerability of older people, this study demonstrated that, compared with older people living in institutions, older people living with families have lower levels of comprehensive health vulnerability, physical health vulnerability, and mental health vulnerability. Some scholars believe that living with family members has a positive effect on the health of older people. Older adults who live with their families have access to financial and emotional support that can help reduce the risk of depression and promote good health [36, 37]. Older people who do not live with their children report poorer self-rated health and higher rates of disability and loneliness. This study also revealed that the overall levels of health vulnerability, physical health vulnerability, and mental health vulnerability of those living alone are significantly greater. Previous studies have pointed out that older people who live alone have worse health and subjective well-being [38, 39]. Most scholars believe that older people who live alone have a high sense of loneliness and that living alone is positively correlated with depression [20, 21]; this may be influenced by the Chinese cultural concept of “The more sons there are, the more happiness”. Older people who live alone in China are considered a vulnerable group in which life stress is often greater than that of older individuals who do not live alone; thus, their physical health is also poorer. In addition, Jiangsu Province is a region that has launched a family planning policy that has brought about changes in family structure. The province has a high proportion of only children, and only children rarely live with their parents. Therefore, there are many older people who live alone, and their health vulnerability is greater. This study also demonstrated that social participation significantly reduces the comprehensive level of health vulnerability, physical health vulnerability, and mental health vulnerability of older people. Relevant studies have shown that participation in social activities has an intrinsic protective function for the physical and mental health of older people. The active participation of older people in social activities is associated with a lower risk of death and a lower prevalence of chronic diseases [40, 41]. Moreover, participation in social activities can improve the happiness and quality of life of older people [42]; relieve negative emotions, such as depression and loneliness [43]; delay declines in cognitive function of elderly people [44]; and reduce the risk of dementia [45]. The promotion of the participation of older people in social activities is an important part of the “implementation of the national strategy of actively coping with population aging” initiative and is the key to realizing the strategy of active aging and a healthy state in China; it can not only promote the health of older people and help them realize their personal value but also benefit the development of society.

The results of this study concerning the impacts of living patterns and social participation on the health vulnerability of older people in urban and rural areas demonstrate that for urban older people, living with families (compared with living in an institution) reduces the overall level of health vulnerability, physical health vulnerability, and mental health vulnerability. Living alone significantly increases overall health vulnerability, physical health vulnerability, and mental health vulnerability. Jiangsu Province is a relatively developed province. Urban older people there are generally retired workers in enterprises and institutions, their work before retirement generally involves mental labor, sedentary time is long, and physical activity time is short. Older people in cities like to use the internet; after retirement, they are accustomed to sitting still, which is not conducive to their health. Older people who live with their families have a greater sense of stress and responsibility, which encourages them to engage in more physical work and reduces their sitting time and thus the risk of disease [46]. In addition, older adults who live with their families may be more likely to have access to social support, such as emotional and instrumental support, both of which have a very positive impact on their mental health [47–49]. Therefore, older people in urban areas who live with their families have better physical and mental health than those who live alone or in nursing homes. This study also revealed that, compared with living in an institution, living with families reduces the mental health vulnerability of rural older people and that living alone increases their mental health vulnerability. However, the effect of residential mode on the physical health vulnerability of rural older adults was not statistically significant. Most old people in rural areas are engaged in agricultural production activities of different intensities and generally quit the labor market later and sit down for only a short time; thus, their physical health is better and less affected by living patterns. Rural older people value the culture of “filial piety”. Living with family can improve the happiness of rural older people and reduce their loneliness, psychological stress and depression [50]. Therefore, living patterns have a significant effect on the mental health of rural older people. In addition, social participation significantly reduces health vulnerability in both urban and rural older people. It is evident that social participation is a positive factor in promoting the health of urban and rural older people [51, 52].

Further analysis of the interaction between living patterns and social participation demonstrated that the interaction term between living with family and social participation was not statistically significant. However, the interaction term between living alone and social participation is statistically significant. Social participation has a greater effect on overall health vulnerability and mental health vulnerability in older people who live alone than in those who live in institutions; this suggests that social participation is more important for health promotion among older people who live alone. Some studies have shown that older people who live alone have poorer health [22] and that social participation enhances the health of older people [53]. However, few studies have focused on the effects of the interaction between living patterns and social participation on the health of older people. This study demonstrated that social participation has a more significant effect on the health of older people who live alone. Previous studies have noted that regular physical activity is an important intervention for reducing disease risk and promoting health in older people [46]. Older adults who live with their families may be more likely to participate in household work, intergenerational care work, or recreational activities with their families. However, the lifestyle of older people who live alone is relatively simple, with less housework and fewer recreational activities, so it is necessary to increase their physical activity through social participation to promote health. Therefore, to improve their health, older people who live alone should be incentivized to participate in as many social activities as possible, thereby reducing health vulnerability.

The interaction between living patterns and social participation was evaluated in urban and rural areas. The effect of the interaction term of living alone and social participation is statistically significant only for the comprehensive level of health vulnerability and the level of mental health vulnerability of urban older people. It is evident that the impact of social participation on the health of urban older people who live alone is more significant. As mentioned earlier, physical activity interventions are an important means to promote health in older adults. Before retirement, older people in urban areas are mostly engaged in mental work, sitting for a long time, and lacking physical activity. In particular, older people who live alone in cities face no family pressure and are more accustomed to sitting quietly and being alone. Active participation in social activities can help change the “sedentary” lifestyle of older people who live alone in cities, thereby promoting health and reducing vulnerability. Older people who live alone in rural areas are more accustomed to physical activities, and social participation has less of an impact on their health. Therefore, increasing the social participation of urban older people who live alone to effectively reduce their health vulnerability is important. This study also revealed that social participation affects the mental health vulnerability of older adults who live alone in urban areas. Numerous studies have shown that physical activity is closely related to mental health [54, 55]. For example, sedentary behavior has an effect on depression [56]. Older people who live alone in cities are more likely to have psychological problems due to a lack of physical activity and sitting for a long time, and they must participate in social activities to reduce their mental health vulnerability. In summary, older people should be encouraged and guided to participate in society in a subjective manner, and children should provide their parents with appropriate financial help and life care to provide strong spiritual encouragement and material support for their active social participation.

## Conclusion

The results of this study indicate that a certain level of health vulnerability exists among Chinese urban and rural older people. The poor living habits of urban older people increase their vulnerability to physical health risks. Older people in rural areas face greater economic pressure and difficulties in ensuring their own livelihood and supporting their children and are more likely to be exposed to mental health risks. Older people who live with their families, especially in urban areas, have a greater sense of stress and responsibility, which encourages them to engage in more physical work, and reduce their sitting time, and thus their risk of disease. In addition, older adults who live with their families may be more likely to have access to social support, such as emotional support and instrumental support, both of which have a very positive impact on their mental health. Rural older people attach importance to the culture of “filial piety”. Living with family can improve the happiness of rural older people and reduce their loneliness, psychological stress and depression. Older people who live alone are often seen as vulnerable and tend to have more stressful lives and poorer physical health. Social participation has a significant effect on the health of older adults who live alone. Older people who live alone, and who have a relatively simple lifestyle with fewer household chores and recreational activities, have a greater need to increase their physical activity through social participation, thereby promoting health. In particular, for older people who live alone in cities, active participation in social activities can help change their “sedentary” lifestyles, thereby promoting physical and mental health and reducing vulnerability.

This study focuses on the effects of living patterns and social participation on the health vulnerability of urban and rural older people. In fact, many factors affect the health vulnerability of older people. Multivariate statistical analysis can be used to identify which urban and rural communities present greater health vulnerability among older adults. Such information would be useful for the design of public policies.

### Limitations

The real-life health vulnerability of elderly people is the result of a combination of complex factors that must be analyzed using more specific and operational vulnerability indicator systems and empirical models. A more complete indicator system should be established in subsequent studies to analyze and explore the health vulnerability of elderly people in greater depth. In addition, the data collected may present biases that are due to the selection of participants, in particular among vulnerable older adults in disadvantaged urban and rural communities. In future research, more reasonable sample selection methods should be considered.

## Data Availability

The data that support the findings of this study are available on request from the corresponding author. The data are not publicly available due to privacy or ethical restrictions.

## Abbreviations

FI: Frailty index
QOL: Quality of life
PA: Physical activity
GAD-7: Generalized Anxiety Disorder-7 scale
SDS: Self-rating depression scale
